# Cook County Hospital

**Published:** 1879-03

**Authors:** J. H. Salisbury


### ARTICLE VII.

### COOK COUNTY HOSPITAL.

(Reported by J. H. SALISBURY, M.D.)

*A Case of Cancer of the Rectum, in which Colotomy was performed for the Relief of Intestinal Accumulation.*

Sailor, age 35, native of England. Admitted to Cook County Hospital, July 9, 1878.

On admission he gave the following history: About two years ago he noticed that he passed blood with his stools. He went to the Buffalo hospital, and was treated for chronic diarrhœa. He left the hospital after five months, somewhat improved, but the discharge of blood and mucus still continued. From this time until October, 1877, he followed the occupation of a sailor. He was troubled during this time with discharges of blood and slime, and occasionally he had some pain in the bowels. Otherwise he was well. In October, 1877, he entered the Marine hospital, in this city (Chicago). His disease was here diagnosticated cancer of the rectum. He left the hospital after four months, without much improvement. Since that time he has kept himself very quiet, and his symptoms have been somewhat better. He has treated himself with carbolic acid and glycerine locally, as an injection.

For the last week previous to admission, he has had more pain in the abdomen than usual and has lost his appetite. His abdomen has swollen considerably, and he has been troubled as formerly with frequent passages of slime and blood. He states that he has lost in the last two years about fifty pounds in weight. His weight is now about 140, and varies between 140 and 160.

On examination the rectum was found almost obliterated by the projection into it of a hard enlargement of its walls. The caliber of the rectum was so small that not even a catheter could be introduced. The patient was suffering great distress from the distension of the abdomen, having had no passage for nine days. He could eat nothing.

On July 15th, Dr. Gunn performed colotomy, making an incision through the integument about five inches in length, running from a point two inches above the anterior third of the iliac crest obliquely upward and backward. After dividing a few fibers of the latissimus dorsi, the operator found, between that muscle and the peritoneum, just at the edge of the quadratus lumborum, a quantity of loose fat, by the removal of which the colon was exposed. Two ligatures were passed through the bowel, which was divided between them. The edges of the wound were then stitched to the edges of the external incision. Considerable soft fecal matter and gas escaped, and the patient expressed himself much relieved. He recovered readily from the operation. The bowels discharged their contents through the artificial opening usually regularly; occasionally a cathartic was required.

About the middle of August the patient began to complain of great pain in the rectum, and to pass blood and slime again. Aug. 26th, a note is made that he passed blood and strands of dead tissue. Oct. 18th. Has difficulty in urinating, passing only a small stream and with pain. The pain continued to increase, and became so severe that for about three weeks before death he received daily four hypodermic injections of morphia, of ½ grain each. Nov. 2d, he had a chill. For about two weeks before death he had œdema of the left leg. Nov. 11th. After three copious discharges of blood per rectum, the patient expired from the exhaustion of the hæmorrhage.

Autopsy 12 hours after death. Very little fluid in the serous cavities. Heart normal but very anæmic; lungs normal and anæmic; one of them a little œdematous. Lower two-thirds of the peritoneal cavity filled with the enormously distended sigmoid flexure of the large intestine. The whole peritoneal surface, both visceral and parietal, is covered with thousands of fine, white, cancerous nodules, varying from the size of a mere dot to that of a small pea. In the parietal layer of the peritoneum, in the right iliac fossa, is a patch of diffuse, confluent, small white masses.

The finger passes from the artificial opening readily upward, into the descending colon, but not downward into the part below. The gases and other contents in the distended part below do not escape at this point when pressure is made on the gut. The artificial anus is found to open into the colon at the portion not covered by the peritoneum, leaving this membrane untouched. Extensive adhesions between the distended bladder and sigmoid flexure from continuous cancerous growths. The ureters very much distended, especially the right. The pelves of both kidneys likewise distended; kidneys much enlarged from hydro-nephrosis, pale and light-colored patches are found in their tissue which look like cancerous infiltration. The left common iliac vein is obliterated from a point an inch above Poupart's ligament to within an inch of its junction with the other, by cancerous growths from adjacent portions of the parietal peritoneum. The lower part of the pelvic cavity is filled with a general, diffuse, cancerous growth, involving all the viscera and the cellular tissue, forming a firm mass, which has broken down in one place and formed a cavity of the size of two hen's eggs. In the wall of the cavity may be found the opening of one of the branches of the internal iliac artery, from which the hæmorrhage proceeded. The bladder distended, walls hypertrophied, openings of the ureters closed by cancerous infiltration, the prostate enlarged from a similar growth. The liver, spleen and brain were very anæmic, but otherwise normal.

